# Metabolic Profiling as Well as Stable Isotope Assisted Metabolic and Proteomic Analysis of RAW 264.7 Macrophages Exposed to Ship Engine Aerosol Emissions: Different Effects of Heavy Fuel Oil and Refined Diesel Fuel

**DOI:** 10.1371/journal.pone.0157964

**Published:** 2016-06-27

**Authors:** Sean C. Sapcariu, Tamara Kanashova, Marco Dilger, Silvia Diabaté, Sebastian Oeder, Johannes Passig, Christian Radischat, Jeroen Buters, Olli Sippula, Thorsten Streibel, Hanns-Rudolf Paur, Christoph Schlager, Sonja Mülhopt, Benjamin Stengel, Rom Rabe, Horst Harndorf, Tobias Krebs, Erwin Karg, Thomas Gröger, Carsten Weiss, Gunnar Dittmar, Karsten Hiller, Ralf Zimmermann

**Affiliations:** 1 Luxembourg Centre for Systems Biomedicine 6, avenue du Swing, L-4362 Esch-sur-Alzette, Luxembourg; 2 Mass Spectrometry Core Unit, Max Delbrück Center for Molecular Medicine Berlin-Buch, Berlin, Germany; 3 Institute of Toxicology and Genetics (ITG), Karlsruhe Institute of Technology, Campus North, Karlsruhe, Germany; 4 Institute for Technical Chemistry (ITC), Karlsruhe Institute of Technology, Campus North, Karlsruhe, Germany; 5 Center of Allergy and Environment (ZAUM), Helmholtz Zentrum München and Technische Universität München, Munich, Germany; 6 CK-CARE, Christine Kühne Center for Allergy Research and Education, Davos, Switzerland; 7 Joint Mass Spectrometry Centre, Division of Analytical and Technical Chemistry, Institute of Chemistry, University Rostock, Rostock, Germany; 8 University of Eastern Finland, Department of Environmental Science, P.O. Box 1627, FI-70211 Kuopio, Finland; 9 Joint Mass Spectrometry Centre, CMA – Comprehensive Molecular Analytics, Helmholtz Zentrum München, Neuherberg, Germany; 10 Chair of Piston Machines and Internal Combustion Engines, University Rostock, Rostock, Germany; 11 Vitrocell GmbH, Tübingen, Germany; 12 HICE – Helmholtz Virtual Institute of Complex Molecular Systems in Environmental Health – Aerosols and Health, Neuherberg, Rostock, Munich, Karlsruhe, Berlin, Waldkirch, Germany; Kuopio, Finland; Cardiff, United Kingdom; Esch-Belval, Luxembourg; 13 German Center for Lung Research (DZL), Munich, Germany; Technion - Israel Institute of Technology, ISRAEL

## Abstract

Exposure to air pollution resulting from fossil fuel combustion has been linked to multiple short-term and long term health effects. In a previous study, exposure of lung epithelial cells to engine exhaust from heavy fuel oil (HFO) and diesel fuel (DF), two of the main fuels used in marine engines, led to an increased regulation of several pathways associated with adverse cellular effects, including pro-inflammatory pathways. In addition, DF exhaust exposure was shown to have a wider response on multiple cellular regulatory levels compared to HFO emissions, suggesting a potentially higher toxicity of DF emissions over HFO. In order to further understand these effects, as well as to validate these findings in another cell line, we investigated macrophages under the same conditions as a more inflammation-relevant model. An air-liquid interface aerosol exposure system was used to provide a more biologically relevant exposure system compared to submerged experiments, with cells exposed to either the complete aerosol (particle and gas phase), or the gas phase only (with particles filtered out). Data from cytotoxicity assays were integrated with metabolomics and proteomics analyses, including stable isotope-assisted metabolomics, in order to uncover pathways affected by combustion aerosol exposure in macrophages. Through this approach, we determined differing phenotypic effects associated with the different components of aerosol. The particle phase of diluted combustion aerosols was found to induce increased cell death in macrophages, while the gas phase was found more to affect the metabolic profile. In particular, a higher cytotoxicity of DF aerosol emission was observed in relation to the HFO aerosol. Furthermore, macrophage exposure to the gas phase of HFO leads to an induction of a pro-inflammatory metabolic and proteomic phenotype. These results validate the effects found in lung epithelial cells, confirming the role of inflammation and cellular stress in the response to combustion aerosols.

## Introduction

Air pollution from anthropogenic sources are consistently associated with adverse health effects, such as asthma, cardiovascular problems, and cancer [1, 2]. Near harbors and other water-based industrial areas, there is a large amount of exhaust from ship engines, which run on different fuel types with very different chemical compositions. Marine gas oil, or diesel fuel (DF), is similar to the standard fuel used in diesel automobiles, while heavy fuel oil (HFO) has very different chemical properties, including much higher levels of toxic chemicals, such as polycyclic aromatic hydrocarbons, carbonylic compounds, and also different transition metals. Epidemiological studies have associated the combustion of these fuels with an increased incidence of lung and cardiovascular diseases [3], and it is important to study the mechanisms of these effects so that through technology, measures can be taken which focus on minimizing the health problems caused by ship emissions.

A prominent effect of combustion aerosols on human health is an increase of inflammatory responses in affected tissues. As inhalation is the main route of aerosol exposure, the lungs are primarily affected. Many studies have investigated the effects of particulate matter (PM) emissions on lung cells (both *in vitro* and *in vivo* [4–7]), but most of these studies have focused on the use of submerged experiments. While useful for studying the effects of particles present in aerosols, these exposures are less representative of real-world inhalation of combustion emissions. For this reason, exposure at an air-liquid interface (ALI) can be used to represent a more biologically accurate exposure to lung epithelial cells, as well as other cells present in lung tissue. In addition to being a more realistic *in vitro* model system, these experiments are able to elucidate differences between the gas and particle components of aerosols through the use of in-line filters [8]. Validation studies for the ALI system have been performed previously [9, 10], and there have also been multiple studies that have used ALI technology to uncover novel chemical and biological insights, including more accurate modeling of particle deposition efficiencies [11], as well as the investigation of epithelial cell-macrophage co-culture responses to waste incineration emission aerosol [12]. In addition, and not only for the reasons outlined above, ALI systems are preferred compared to submerged experiments for cell exposure, as has been discussed previously [13].

In the framework of the international project HICE (Helmholtz Virtual Institute of Complex Molecular Systems in Environmental Health, www.hice-vi.eu) a novel ALI exposure technology for cell exposure in the field has been established in conjunction with multi-omics cellular response studies. This concept has been applied to study emissions from a ship engine and their effects on a lung epithelial cell line [14]. To summarize the previous study, biological effects triggered by PM from ship diesel engines in a lung epithelial cell line pointed out that particles originated from the use of refined, sulfur-free DF induce a similar or even stronger acute activation of many adverse pathways than the use of HFO [14]. This was a surprising result, as PM from HFO contains much higher concentrations of known toxic compounds, including polycyclic aromatic hydrocarbons (PAH), oxygenated compounds and transition metals, such as vanadium and nickel [14, 15]. In addition, one of the main pathways affected was the inflammatory response, promoting increased transcription of pro-inflammatory markers, including IL-8, IL-1, and CXCL2, particularly for HFO exposure. It was also hypothesized that endocytosis of particulate matter was regulated in response to both fuel types. Transcriptomic and proteomic effects highlighted that energy metabolism was affected as well, suggesting a focus on metabolism would be interesting for this study. In order to extend the results of the previous study to a more inflammatory-focused and phagocytotic model, as well as to compare the effects of DF and HFO exposure in another cell type to validate the previous findings, we used the same experimental setup to simultaneously investigate a macrophage cell line using a primarily metabolism-focused approach, supported by quantitative proteomics (SILAC) and cytotoxicological measurements.

Macrophages are a type of differentiated phagocytotic monocyte that have important roles in both innate and adaptive immunity, and particularly in the inflammatory response [16]. There is a large heterogeneity in macrophage phenotypes (largely related to their activation states) [17], and these cells play an important role in maintaining tissue homeostasis, mediating pro- and anti-inflammatory reactions, as well as a variety of other well-characterized functions [18]. In lung tissue, macrophages are present in much higher amounts than other immune cells [19], and populations can increase under inflammatory conditions (along with the associated pro-inflammatory phenotypic changes) [20].

The effect of combustion aerosols on the induction of inflammation in macrophage populations has been studied both in ALI experiments and in animal studies [12, 21–23]. While much of the research has focused on cytokine production, transcriptional expression, and physiological effects (such as changes in immune cell populations) involved in aerosol-induced acute lung inflammation, metabolic changes due to these conditions have been largely unexplored. Where metabolism has been mentioned, it has been tied to enzymatic activity or transcriptional regulation of metabolic genes and proteins. Metabolomics studies focus on the metabolic profile in the cell, looking at changes in the effector molecules in the various biochemical pathways that control cell function [24]. Non-targeted metabolomics analyses provide a comprehensive view of metabolites present in a biological system, while stable isotope-assisted metabolomics makes use of labeled tracer molecules to elucidate the flux of biomolecules through central carbon metabolism [25]. In addition, metabolomics data can be integrated with other data to uncover a more complete picture of biological function [26].

In this study, we expose a murine macrophage cell line to diluted emission aerosols from a ship diesel engine operated with two types of marine fuel: DF and HFO, and study the cytotoxic, metabolic, and proteomic effects. DF is commonly used in inland waterway transportation, while HFO and DF are both used in sea-based transportation. The study mimics a real-life engine load scenario, with both a full aerosol exposure and a particle-filtered aerosol exposure (with only the gas phase exposed to the cells), in order to look at the net effect of the particles. The gas phase of these aerosols is characterized, and cellular effects are presented from a metabolomics point of view, with integration of complementary data from toxicological and proteomic studies. Using this approach, we were able to highlight the metabolic changes and some proteomic effects associated with the inflammatory response of macrophages to aerosol exposure. Furthermore, the current work aims to verify the findings of the previous study on lung epithelial cells, in order to add support to their conclusion that DF PM exposure may have similar or possibly stronger biological effects than HFO exposure at comparable deposition doses.

## Materials and Methods

### Engine and exposure

A four-stroke single-cylinder direct-injected diesel engine test bed situated at the University of Rostock in the Chair of Piston Machines and Internal Combustion Engines was used to generate aerosol for the exposure study. Heavy fuel oil HFO 180 was used as a representative fuel for ship operation outside of sulfur emission control areas (SECAs). Distillate diesel fuel (DF) according to DIN EN 590 was used as a reference. The DF fuel represents a modern, sulfur-free distilled fuel as used in inland waterway transportation and SECAs. The engine ran at four different operating points: 100%, 75%, 50%, and 25% load at a nominal speed of 1,500 rev/min. A detailed characterization of the aerosol composition at the ALI exposure system was performed, [27]. The duration of each operation point was set in accordance to their weighting factors as described in ISO 8178-4 E2. The total cycle duration was 2 hours, and this cycle was run twice for a single exposure. To obtain comparable particle deposition doses for the experiments with the two fuel types, clean air dilution ratios of 1:40 and 1:100 were used for the exhaust aerosols of DF and HFO emissions, respectively. The diluted concentrations of PM 2.5 in the aerosols were 340 μg/cm^3^ and 760 μg/cm^3^ for DF and HFO, respectively. Based on these values, and a post-experiment gravimetric filter analysis of PM 2.5 and assuming a constant deposition probability of 1.5%, which was determined using previous measurements from ALI exposure systems [11], the particle mass deposited on the lung cell monolayer surface was calculated at 28 ± 1.5 ng/cm^2^ (DF) and 56 ± 0.7 ng/cm^2^ (HFO) for the 4 hour exposure [14].

### Physical and chemical profiling of aerosols

Profiling of the particulate matter from the exposure aersosols from both fuel types was performed as in the previous study [14, 15]. To summarize, on-line and off-line analysis techniques, including gas chromatography mass spectrometry, high resolution mass spectrometry (ESI-FTICR-MS), energy-dispersive X-ray spectroscopy (EDX), on-line aerosol mass spectrometry, well as others have been used. Detailed information on the particulate matter analysis has been reported previously [14], and a summary is shown in S1 Fig. On-line analyses were performed in parallel with cellular exposures, and off-line techniques were performed using particulate matter collected on filters during cellular exposures.

Data on gas phase aerosol compounds were derived from on-line photo-ionization mass spectrometry [28]. Aromatic species were measured by resonance enhanced multi-photon ionization (REMPI) mass spcectrometry, which utilizes intense short laser pulses of 266 nm generated by a Nd:YAG laser. Ionization occurs by subsequent ionization of two photons, providing selectivity and enhanced sensitivity for aromatic species. Benzene and butadiene were analyzed by on-line single photon ionization (SPI) mass spectrometry using 126 nm Vacuum-UV radiation generated by an electron beam pumped rare gas excimer lamp [29]. Ionization occurs by absorption of a single photon. Carbonyl compounds were sampled by derivatisation with 2,4-dinitrophenylhydrazine (DNPH) using commercially available cartridges ‘ORBO/555’ (Sigma Aldrich, USA) and subsequently analyzed by GC-MS [27]

### Cell culture conditions

The mouse macrophage RAW 264.7 cell line was obtained from ATCC (ATCC^©^ TIB-71™). Cells were cultured in RPMI-1640 medium supplemented with 10% (v/v) Fetal Bovine Serum and 100 U/ml penicillin, 100 mg/ml streptomycin (Life Technologies, Darmstadt), and cultivated in an incubator at 37°C with 5% CO_2_. For stable isotope labeling experiments, cells were seeded 24 hours before the experiment in RPMI-1640 medium as above, with 12.5 mmol/L U-^13^C_6_-Glucose (Cambridge Isotope Laboratories, USA) substituted for unlabeled glucose.

### Air-liquid interface exposure

An automated ALI exposure system station (VITROCELL Systems, GmbH, Waldkirch, Germany) with 18 exposure positions was used as the interface for cellular exposures of the diesel engine exhaust [30]. ALI exposures were performed in a custom built mobile HICE S2 bio safety laboratory, placed next to the engine hall. Diluted DF or HFO aerosol was led through heated stainless steel lines from the engine test bed into the ALI system. The combustion aerosols coming from the engine’s exhaust pipe were cooled and diluted with sterile air, and then transferred directly to the ALI for cellular exposure. A PM 2.5 impactor removed large particles before the exhaust entered the ALI system. Cells exposed to complete aerosols used the humidified exhaust from the engine, while cells exposed to only the gas phase had particles removed directly above the cell chamber using a Whatman HEPA polydisc filter (GE Healthcare). Exposures were performed as described for ALI exposures of BEAS2B and A549 epithelium cells [14].

Cells were seeded in FCS supplemented RPMI-1640 on transferrable 24 mm Transwell^®^ inserts with a 0.4 μm pore polyester membrane (Type #3450, Corning, NY, USA) 24h before exposure at a density of 1 x 10^6^ cells/mL/insert (2.1 x 10^5^ cells/cm^2^ growth area) with 1.5 mL cell culture medium provided beneath the insert membrane. For cell exposure, the culture medium on the apical side was completely removed and cells were placed in the ALI exposure system with RPMI-1640 medium without FBS, supplemented with 10 mM HEPES, provided at the basolateral side. Cells were then exposed for 4h to the diluted and conditioned (85% r.h., 37°C, maintained by a software controlled humidifier in the ALI exposure system) aerosols with a controlled flow of 100 mL/min for each insert [13, 30]. Cells were exposed to both the complete aerosol (with both particle phase and gas phase), and the gas phase only (with particles filtered out of the complete aerosol by a high efficiency particle membrane filter). All fuel-specific results represent both the filtered and unfiltered treatments, unless otherwise stated.

### LDH release assay

After exposure, medium from the compartment under the membrane was collected and frozen at -80°C for later analysis. An aliquot was used for quantification of released lactate dehydrogenase (LDH), an indicator of plasma membrane integrity. An LDH detection kit was used in accordance with the manufacturers’ instructions (Roche, Mannheim, Germany) with slight modifications: the dye solution was diluted 1:1 (v/v) with PBS to slow down the reaction time caused by elevated LDH values due to the high cell densities used for ALI exposure experiments. After 20 minutes, before saturation was reached, the reaction was stopped and the absorbance of the reaction mix was measured at 490 nm with a microplate reader. Absorbance read from the samples was normalized to the absorbance of blank medium. Statistical analysis was performed using an analysis of variance (ANOVA) test, followed by a post-hoc Tukey test for pairwise statistical analysis. Cells exposed with HEPA-filtered ambient air humidified to 85% r.h. at 37°C used as a control treatment. Cells used for a positive control for cellular toxicity were kept under control conditions and lysed with Triton X-100 (Sigma Aldrich) for 30 minutes prior to the end of the exposure period.

### Metabolite extraction and GC-MS processing

For metabolomics experiments, cells kept in an incubator at 37°C with 5% CO_2_ were used as a control treatment. Metabolite extraction was performed as previously described [31]. Briefly, after washing cells with 0.9% NaCl, a 1:1:1 extraction using methanol, water, and chloroform was used to quench metabolism and create a three-phase separation. Cells were agitated for 20 minutes and then centrifuged to enforce phase separation. 200 μL of the polar phase was used for gas chromatography coupled to mass spectometry (GC-MS) analysis. The interphase was then used for proteomics analysis.

Compound derivatization was performed with a Gerstel autosampler directly before measurement on the GC-MS. Dried metabolites were dissolved in 15 μL of 2% methoxyamine hydrochloride in pyridine at a temperature of 40°C for 30 minutes. Then, 15 μL of 2,2,2-trifluoro-N-methyl-N-trimethylsilyl-acetamide + 1% chloro-trimethyl-silane was added and incubated at 40°C for 30 minutes. Methoxyamine hydrochloride derivatization breaks apart cyclic forms of some metabolites, reducing confusing of metabolites with different forms (such as glucose) eluting at different times based upon their structure. 2,2,2-trifluoro-N-methyl-N-trimethylsilyl-acetamide + 1% chloro-trimethyl-silane is used to block polar groups and allow for efficient transition of the metabolites into the gas phase.

The metabolite extracts were measured on an Agilent 7890 GC with a 30 m DB-35MS capillary column (Agilent Technologies) with an internal diameter of 0.25 mm and a film of 0.25 μm. The GC was connected to an Agilent 5975C MS operating in electron ionization (EI) at 70 eV. The MS source was kept at a constant temperature of 230°C and the quadrupole at 150°C. The detector was operated in scan mode with an m/z range of 70 to 800.

1 μL of derivatized sample was injected at 270°C in splitless mode. Helium was used as the carrier gas at a flow rate of 1 mL/min. The GC temperature program started at 80°C with a hold for 6 minutes, followed by a gradient of 6°C/min to 300°C, a hold for 10 minutes, and an additional gradient of 10°C/min to 325°C. The total splitless GC-MS run time of one sample was 59 minutes. An alkane mix was run with the experimental sequence in order to provide retention index calibration for the experimental samples. The above program was used for the measurement of polar intracellular metabolic extracts from samples. In order to determine secretion of lactic acid, both control and experimental cellular medium was analyzed with the above parameters in a 25 minute method.

All chromatograms were normalized to a standard alkane mix (Sigma 68281) to standardize retention times (RT) to retention indicies (RI) across sample runs. To identify metabolites found in experimental chromatograms, an in-house developed metabolite library was used, and mass spectra as well as normalized RI values were compared. This library was created by running analytical standards run on the same instruments using the same parameters as our experimental samples. All identified metabolites were verified using the NIST 11 spectral database [32]. Metabolic analysis and mass isotopomer distribution (MID) calculation, including correction for naturally occuring stable isotopes, was performed with the MetaboliteDetector software [33, 34].

Metabolic data were factor-normalized based on standardized pool samples run with each GC-MS sequence. The pool samples were a standardized extract of intracellular metabolites from control conditions, to provide a baseline and control for GC-MS drift and buildup on the GC column. Changes in the pool samples were calculated, and this factor was applied to normalize the experimental samples. In order to reduce noise and to focus on metabolites changing between conditions, a further filtering was applied, removing all compounds present in less than 80% of all treatments. All metabolic statistical significance measurements were performed using an unpaired, two-tailed student’s t-test unless otherwise noted.

### Stable isotope labeling by amino acids in cell culture (SILAC)

RAW 264.7 mouse macrophages were cultured for six passages as above, with either 48.67 μg/mL H4-lysine (lysine 0, Sigma-Aldrich) or D4-lysine (lysine 4, Sigma-Aldrich) to achieve complete labeling of the proteome [35]. In order to detect unlabeled contaminants for each sample the reverse experiment was performed by exchanging lysine 0 and lysine 4.

### Proteome extraction and LC-MS/MS analysis of peptides

Proteome extraction was performed as previously described [31]. In brief, after metabolite extraction as above, the proteins were present in the interphase of the sample. The tertiary and secondary structure of the proteins were broken down with TCEP and chloroacetamide, and LysC protease was added to digest the proteins. After this, the samples were used for liquid chromatography coupled to two-dimensional mass spectrometry (LC-MS/MS).

The protein extracts were digested using an automated sample-preparation workflow [36]. The purified peptides were lyophilized and resuspended in 0.06% FA/3% ACN buffer and separated on an in-house packed reversed-phase chromatography column (20 cm length, 75 μm ID, 3 μm—Dr. Maisch C18). A 155 min gradient (solvent A: 5% acetonitrile, 0.1% formic acid; solvent B: 80% acetonitrile, 0.1% formic acid) was applied for the samples. A volume of 5 μL sample was injected and the peptides eluted with gradients of 4 to 76% ACN and 0.1% formic acid in water at flow rates of 0.25 μL/m. The samples were injected into a Q-Exactive Orbital-trap mass spectrometer (Thermo-Fisher GmbH, Germany) using electrospray ionization at spray voltage of 2.2 kV and measured twice in a data-dependent acquisition mode, selecting the top 10 peaks for HCD fragmentation. MS acquisition was performed at a resolution of 70,000 in the scan range from 300 to 1700 m/z. Dynamic exclusion was set to 30 s and the normalized collision energy to 26eV. The mass window for precursor ion selection was set to 2.0 m/z.

### Proteomics Data Analysis and Bioinformatics

The recorded MS-files were analyzed using the MaxQuant software package (version 1.2.2.5) with the IPI-mouse database (version 3.84) complemented with frequently observed contaminants using oxidation and acetylation as variable modifications and carbamidomethylation of cysteins as fixed modification with a maximum of 2 missed cleavages.

Data obtained from the mass spectrometric data was log-transformed for the Aerosol/Gas replicates. Means for the four replicates were calculated and used for the determination of significantly regulated proteins. The cut-off for the experiments was set to 10% for the up- and downregulated proteins. The regulated proteins were used for the Gene Ontology analysis using the DAVID online tool [37].

## Results

### Chemical and physical properties of the ship engine exposure aerosol

**Fig 1:**
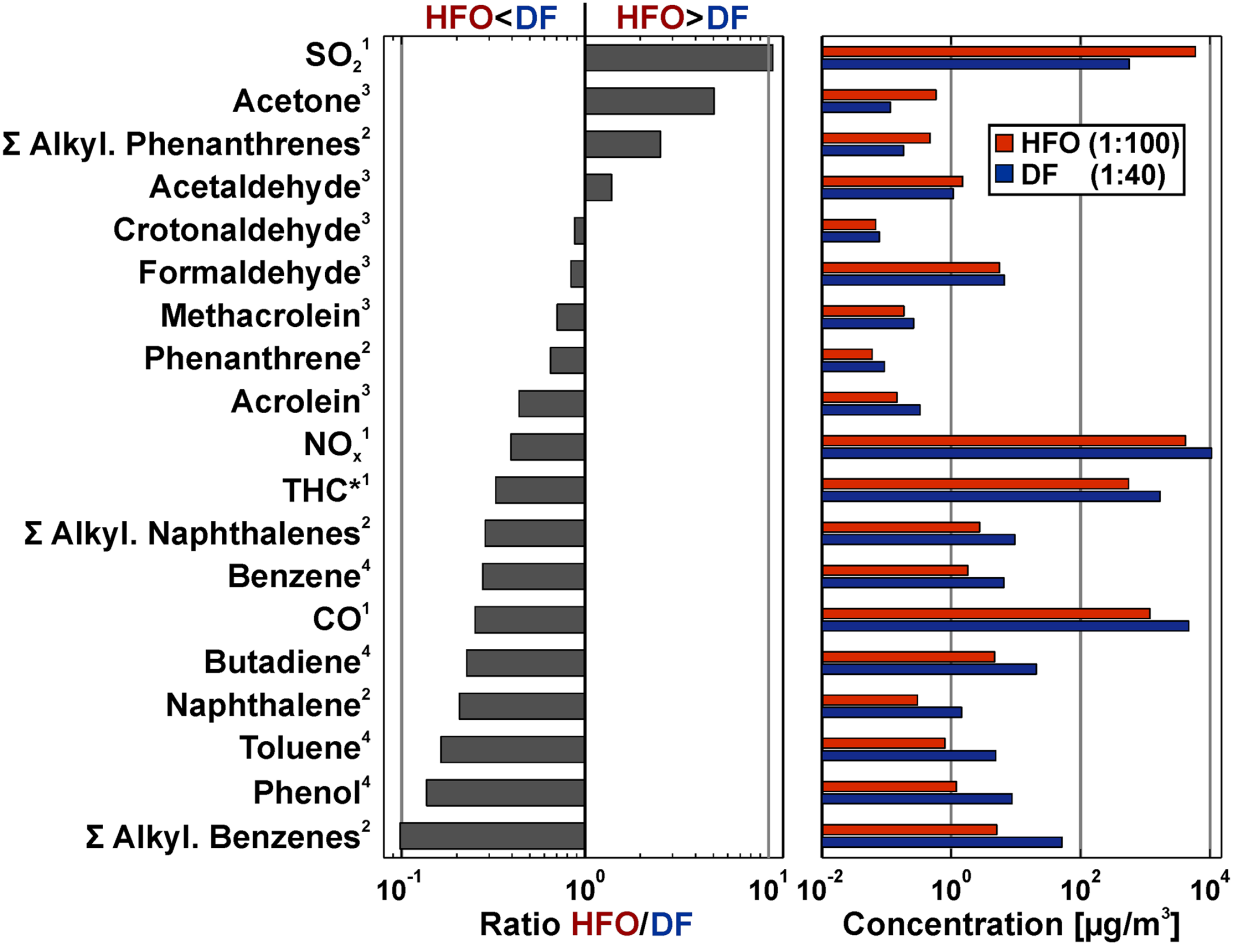
Characterization of gas phase from HFO and DF aerosol (cell exposure concentrations including dilution). The concentrations of each component measured in the aerosol are shown on the right, while the concentration ratios are shown on the left. Exponents refer to method used: (1) Gas monitor, (2) On-line photo ionisation mass spectrometry (REMPI-MS), (3) Gas chromatography mass spectromery, (4) On-line photo ionisation mass spectrometry (SPI-MS).

In order to understand the composition of the aerosols that the cells were exposed to, analysis of the chemicals in both the particulate matter (PM) (see ref. [14] and S1 Fig for a summary) and the gas phase (new data presented in this work in Fig 1) of ship engine emission from both fuels was performed. Note that the data presented in S1 Fig and Fig 1 are representing the exposure concentrations as subjected to the cells in the ALI systems (thus including the different dilution ratios that were applied to generally equalize the PM concentrations). The physical characterization (PM size distribution and mass) has been reported in detail previously [14]. This information, along with the new data on the gas phase composition (Fig 1), has been used to obtain as well as estimate and validate the estimated exposure dose for HFO and DF PM (see above). In the following, the previously reported chemical composition of the particulate matter is summarized and the here newly reported gas phase composition results of the exposure aerosol are discussed.

The PM of the HFO exposure aerosols exhibits much higher concentrations of organic compounds compared to the DF PM. This includes the carcinogenic polycyclic aromatic hydrocarbons as well as aliphatic compounds, such as alkanes (S1 Fig). Investigations with modern high-resolution analytical methods, such as comprehensive two-dimensional gas chromatography with ultra-high resolution mass spectrometry, depict that HFO PM also exhibits a largely increased chemical complexity, as well as considerably higher amounts of toxic transition metals, such as vanadium, nickel and iron [14]. Conversely, the PM of DF aerosols contains more “carbonaceous soot,” measured either thermo-optically as elemental carbon (EC) or optically as black carbon (BC) [15]. The reduced EC and BC values (“soot”) in HFO exhaust is likely due to the high sulfur content of the HFO fuels, which quenches the formation of the larger aromatics and therefore also of soot in the combustion process [38]. In the previous study, the relatively higher content of rather pure carbonaceous soot in the DF PM was associated with the surprisingly high biological activity of DF PM. The results of the comprehensive gas characterization of the exposure aerosol are depicted in Fig 1. With the applied dilution differences, many gas phase compounds (including the bulk pollutants CO and NO) are 2 to 3 times more abundant in the DF exposure aerosol compared to HFO. One of the main differences is that the sum of the alkylated benzenes are found at higher concentrations in DF aerosol while the alkylated phenanthrenes (three-ring PAHs) are considerably more abundant in the HFO aerosol (Fig 1). These compounds are partially unburnt fuel residues and the differences are due to the different boiling point cuts in DF and HFO generation [15]. Smaller alkylated PAHs are currently suspected to be biologically active and potentially health relevant [39–41]. Carboxylic compounds in sum are about equally abundant in HFO and DF aerosol, with acetaldehyde and acetone being more concentrated in the HFO aerosol, while formaldehyde and other low abundant aldehydes are more abundant in the DF aerosol. Furthermore, the content of SO_2_ is very high in the HFO aerosol compared to DF. The SO_2_ concentration, however, is below the concentration where adverse effects are expected, which was determined in the previous study to be 2 ppm for transcriptomics, and 3.3 ppm for LDH release [14].

### Toxicity of aerosol exposure to macrophage cells

**Fig 2:**
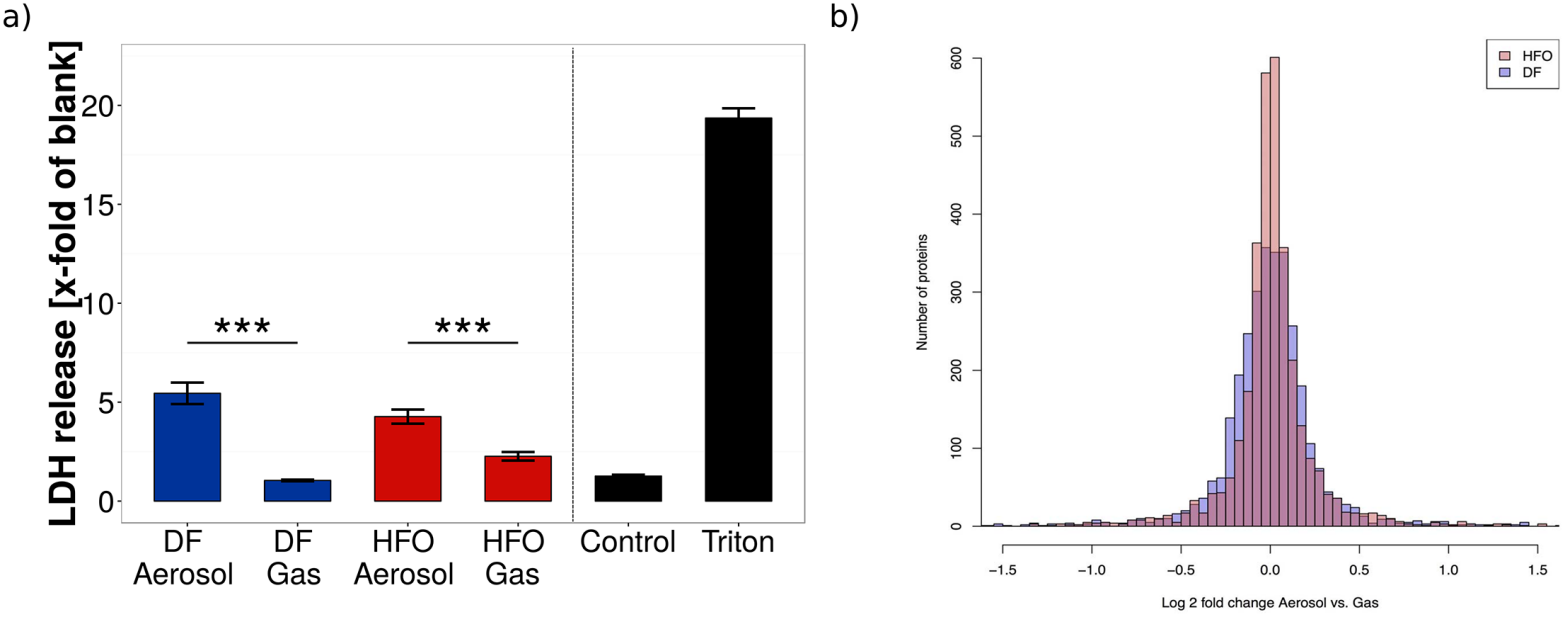
Toxicity of aerosol exposure on macrophages occurs with exposure to both fuel types. (a) Lactate dehydrogenase (LDH) assays were performed on cellular medium after exposure to complete aerosol and filtered aerosols (without particles). DF dilution was 1:40, while HFO dilution was 1:100 in order to obtain comparable particle levels. Blank represents cell free medium, used as a negative control. Triton represents cells lysed with Triton, used as a positive control. Levels represent the mean of three biological replicates (*** = p < 0.001. Error bars represent s.e.m.) (b) Density plot of Log2 fold change distribution of regulated proteins of RAW 264.7 cells in response to DF and HFO particles. RAW 264.7 cells show broadening of the protein regulation in response to DF particles (blue) in comparison to HFO particles (red), which indicates higher amount of regulated proteins in DF treated samples.

DF and HFO aerosol (1:40 and 1:100 diluted, respectively) exposure both led to a detectable loss of membrane integrity due to cell death after 4 hours, with a slightly higher toxicity for the DF aerosol (Fig 2). When the particulate phase was removed from the aerosol through filtering, resulting in an exposure of the cells to the combustion gases only, the toxicity clearly decreased and no significant difference to either the controls or the cell-free “blank” medium could be observed.

The clear reduction of released LDH after particle removal indicates a predominantly particle mediated toxicity for DF aerosol exposure. For HFO aerosol exposure, the gas phase (filtered aerosol) shows some toxicity, although a 2.5 times larger dilution was set for the HFO emissions in order to establish comparable particle deposition doses. This suggests that the HFO gas phase shows more acute cytotoxicity than the DF gas phase, which might be attributed to the higher concentration of gaseous toxic compounds, such as gaseous aldehydes and ketones [27] or alkylated polyaromatic species in the HFO exhaust [39–41]. Thus, with the tested dilution ratios, the gases formed by the DF are not, or in the case of HFO combustion to a part, responsible for the observed aerosol toxicity. The main toxicity, however, is governed by the particle exposure, and it is likely that DF particles show even a stronger cytotoxic impact compared to the HFO particles. These results are in line with our previous study on lung epithelial cells, in which DF particle exposure was shown to produce larger biological effects than HFO particle exposure at comparable deposition doses [14]. These findings are surprising, as the concentration of known toxic compounds (such as PAH, oxygenated species, or heavy metals) is much higher in HFO particles than DF particles. Note that LDH release is a rather strong cytotoxicity endpoint, reflecting irreversible cell death by membrane damage. A lower LDH-based toxicity, therefore, does not exclude the presence of strong adverse cellular effects.

Results from the proteomics analysis show that particles from DF ship diesel combustion aerosols cause broader proteomic response in RAW 264.7 cells than particles from HFO ship diesel combustion. Fig 2 shows a larger distribution of proteins that are differentially regulated in response to DF in comparison to HFO, which indicates higher amount of up and down regulated proteins in DF combustion treated samples. This result is very similar to the previous observations in lung epithelial cells [14], and reinforces this finding in a further lung-relevant cell type. The broadening of protein regulation does not itself prove increased toxicity, but instead shows a wide biological reaction to the given aerosol exposure conditions. In conjunction with the cytotoxicity data, the proteomics results support for the conclusion that DF particle exposure leads to a higher cytotoxic effect in macrophages compared to the HFO particle exposure, likely due to the relatively high soot content of the DF aerosol.

### Differential metabolic profiles of aerosol-treated macrophages

**Fig 3:**
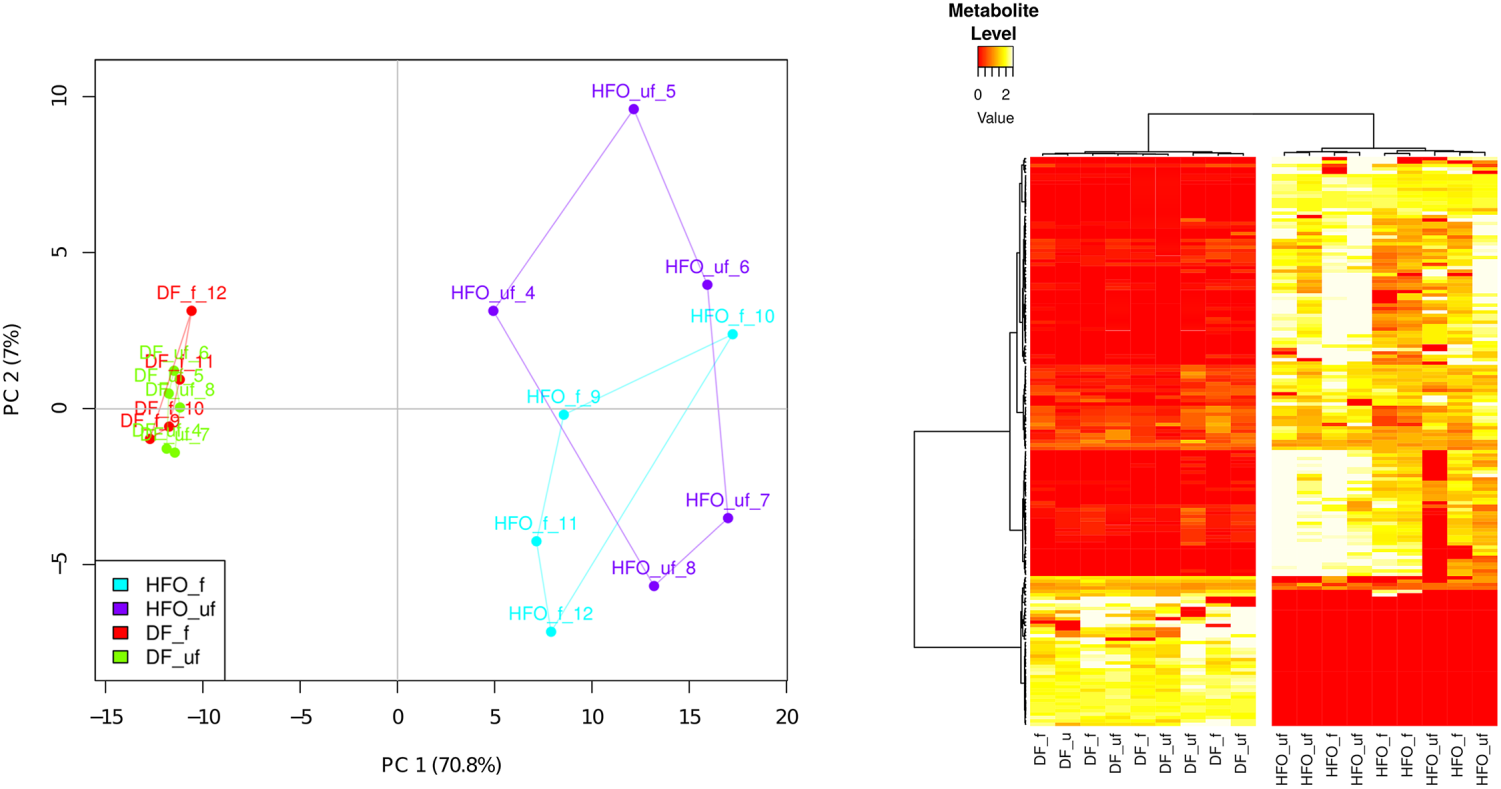
Metabolic profile of macrophages exposed to combustion aerosols. (a) Principal Component Analysis shows separation of fuel types, but no separation by presence of particles. (b) Heatmap represents metabolites with significantly differing abundances between treatments (ANOVA test, p < 0.01). Data was batch normalized, and metabolites found in less than 80% of all treatments were removed. DF: Diesel Fuel, HFO: Heavy Fuel Oil, f: filtered aerosol exposure (n = 4), uf: unfiltered (complete) aerosol exposure (n = 5).

Non-targeted metabolomics analysis uncovered 230 compounds present in the four different conditions. After normalization and filtering out compounds present in less than 80% of each treatment, 203 compounds were subjected to principal component analysis (PCA) and ANOVA analysis. PCA analysis shows groupings based upon fuel type (70.8% of explained variance), with minimal separation due to particle phase (7% of explained variance) (Fig 3). This result is the opposite of the toxicity profile, suggesting that metabolic effects are due to the gas phase of the aerosol more than the PM.

After ANOVA analysis, 167 compounds were found to be significantly differing between HFO and DF exposure at a cutoff of p < 0.01. Of the compounds discovered, 34 were able to be identified with our in-house metabolite library, and are listed in S1 Table. Clustering of significantly changed metabolite groupings show almost no separation between complete aerosol and gas phase exposure (Fig 3). This suggests that the effect of the particulate matter on the metabolic profile of macrophages is negligible, compared to the gas phase. Most compounds were found at increased levels with HFO exposure, while relatively fewer compounds (including Tyrosine and glycerol) are increased under DF treatment.

**Fig 4:**
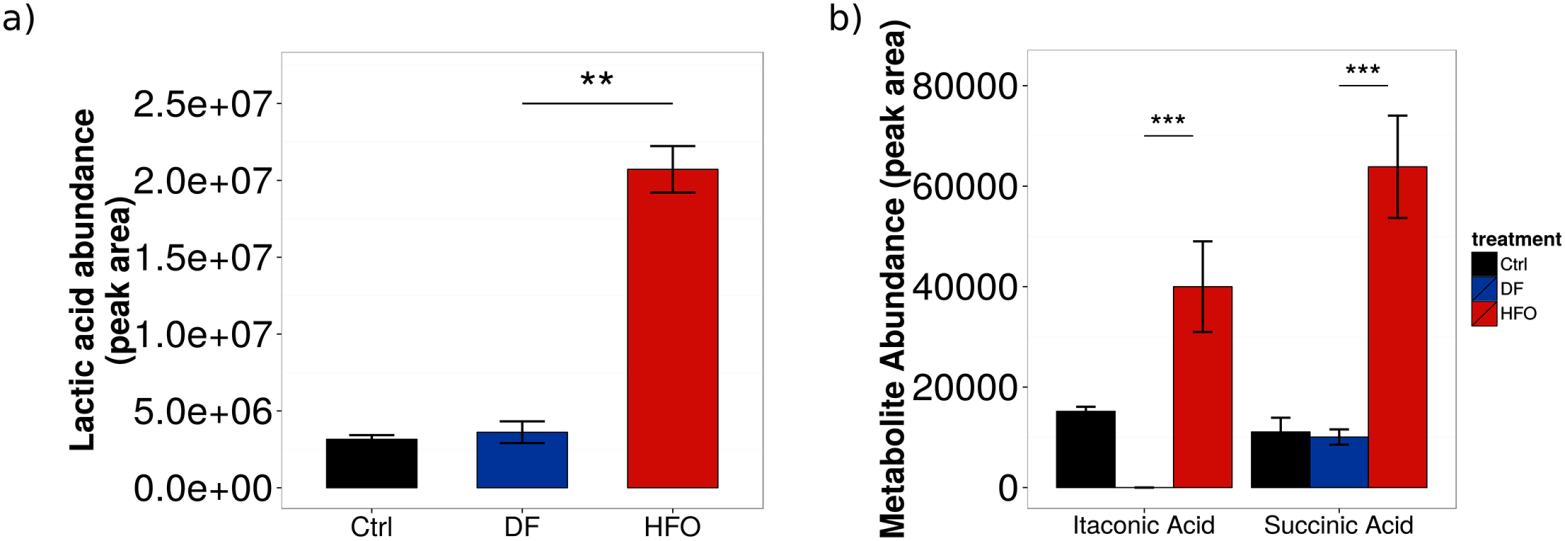
Levels of intracellular metabolite pools in aerosol-exposed macrophages. Pools of (a) lactic acid, (b) succinic acid, and itaconic acid were measured. Levels represent the mean of 9 biological replicates. *** = p < 0.001. Error bars represent s.e.m.

Certain metabolites found increased in HFO-exposed macrophages suggest a pro-inflammatory metabolic phenotype, specifically succinic acid and lactic acid (Fig 4). Succinic acid levels have been found to be increased in pro-inflammatory macrophages to aid increased inflammatory cytokine production [26]; this has been shown to lead to an increased glycolytic flux with reduced pyruvate intake into the TCA cycle [42]. Increased glycolysis with decreased pyruvate flux into the TCA cycle would shunt the flux towards lactic acid. This cellular metabolism profile is common in certain cellular conditions, and is often called the Warburg effect [43]. The Warburg effect is mostly associated with tumor cells, however has been recently found to be present in pro-inflammatory macrophages. [44]

Another metabolite, itaconic acid, has been associated with antimicrobial activity of pro-inflammatory macrophages [45]. Itaconic acid has only been found in certain immune cells (including macrophages), and only when these cells were in a pro-inflammatory state. Therefore, it is a good marker for inflammatory macrophages. To identify this metabolite, experimental spectra were compared to an itaconic acid standard measured previously and integrated into our metabolite identification library (S2 Fig). Itaconic acid was found in all macrophages exposed to HFO aerosols, but was not present in DF-treated macrophages (Fig 4), a result independent of the presence of PM therefore an effect of gas phase exposure. Some itaconic acid was also found in the control cells, at higher levels than DF treatment. The DF treated cells have a much more complicated chromatogram, and this would increase the noise baseline for the analysis. The itaconic acid signal might fall below the baseline, and would therefore be not measured even if it is present in low amounts. However, its presence in macrophages stimulated with aerosols could point to a novel role for this metabolite in inflammation-governed environmental health effects.

The metabolic profile of HFO-exposed macrophages also shows increased levels of other compounds compared to DF-exposure. The increase of adenine and uracil, two bases involved in nucleic acid synthesis, suggests increased cellular DNA and RNA production, a process known to be increased in activated macrophages [46]. Adenine is also an important molecule for energy metabolism, present in both ATP and NAD^+^; its increase could be tied to an increased cellular energy production from increased glycolytic flux to lactic acid (which produces ATP, and aids in NAD+/NADH balancing), necessary for macrophages dealing with inflammatory stimuli.

### Effects of aerosol treatment on metabolic dynamics: Carbon flux from glucose to intracellular metabolites

Stable-isotope labeling provides a complementary source of information on how glycolytic flux is impacted by combustion aerosol exposure. By looking at the labeling pattern of certain metabolites in cells cultured with uniformly ^13^C-labeled glucose, we were able to gain insight into how the glucose is metabolized in the cell through central carbon metabolism, and the rates of glycolysis and glucose oxidation can be compared between different conditions. We measured the labeling in lactic acid, giving more information into glycolytic flux, as well as two metabolites from the TCA cycle (fumaric acid and glutamic acid), which allows the tracing of how glucose is differentially oxidized through the TCA cycle with exposure to the different combustion aerosols.

**Fig 5:**
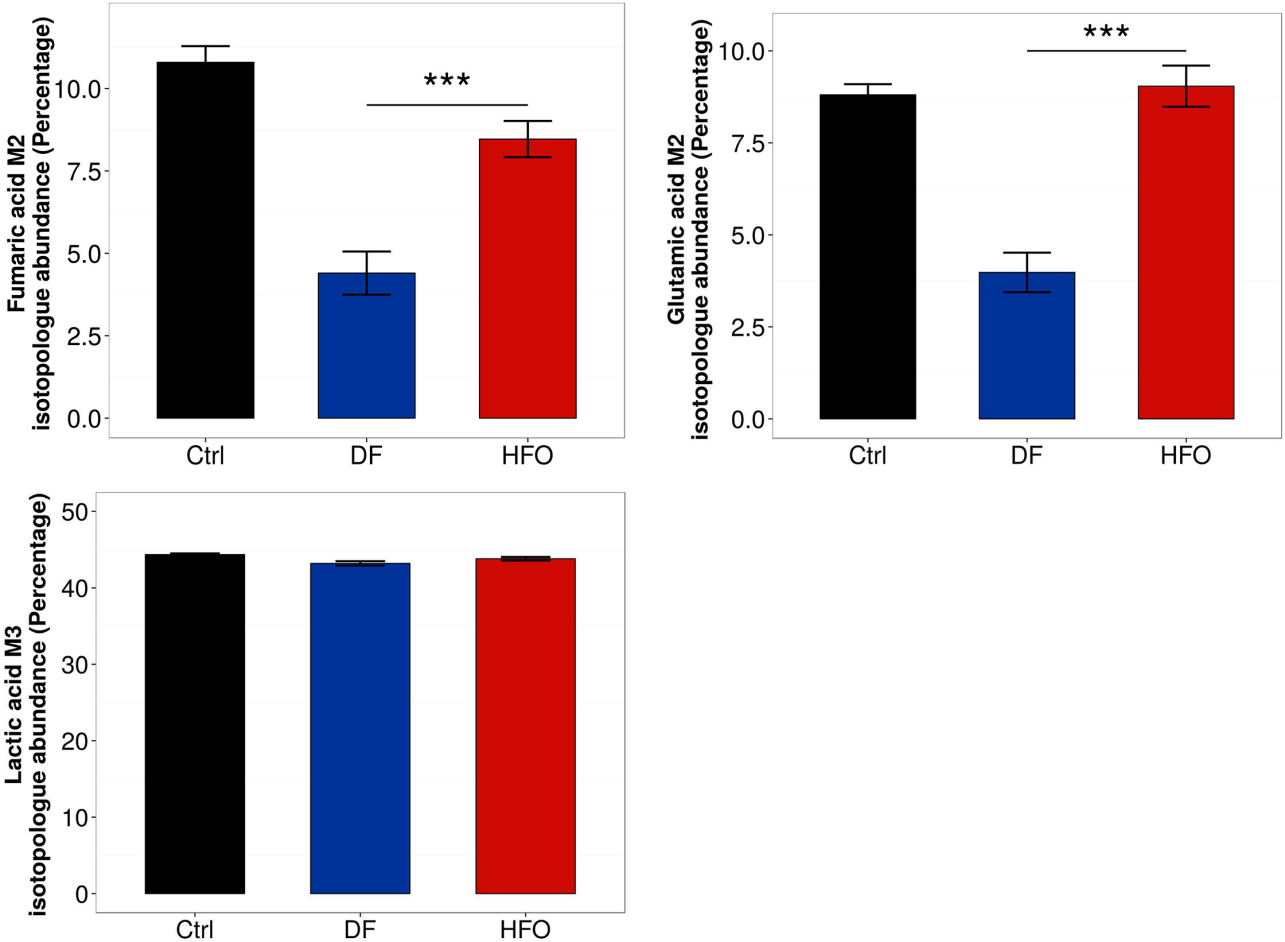
Relative oxidation of glucose in the TCA cycle for aerosol-exposed macrophages. Metabolism of U-^13^C_6_-Glucose was measured in macrophages after exposure. (a) M2 fumaric acid isotopologue levels, (b) M2 glutamic acid isotopologue levels, and (c) M3 lactic acid isotopologue levels. Levels represent the mean of 4–6 biological replicates. *** = p < 0.001. Error bars represent s.e.m.

While both aerosol types lead to a similar amount of lactic acid labeling derived from glucose (Fig 5), labeling of TCA cycle intermediates derived from glucose were significantly reduced in DF exposure compared to HFO exposure (Fig 5). These results suggest that DF-exposed macrophages have a decreased amount of relative glucose oxidation into the TCA cycle compared to macrophages with HFO-exposure. As HFO stimulates a pro-inflammatory phenotype in the macrophages, this decrease in glucose oxidation is presumably tied to an alternative metabolic phenotype. As it is known that macrophages exhibit a wide variety of phenotypic states depending on the stimulant [47], the metabolic changes seen here suggest a macrophage state slightly different from canonical activation states.

### Effects of combustion aerosol treatment on the proteome in RAW 264.7 macrophages

Analysis of the changes to the proteome of exposed RAW 264.7 cells support the findings of the metabolomics analysis, with differing regulation of proteins by HFO particles compared to DF particles (Fig 2 and S3 Fig). An analysis for gene set enrichment of the regulated proteins using the DAVID online pathway analysis tool [37] showed that different pathways and biological processes are induced in response to DF and HFO particles. Two of the main pathways seen to be affected by PM exposure are endocytosis and the activation of the immune response.

**Fig 6:**
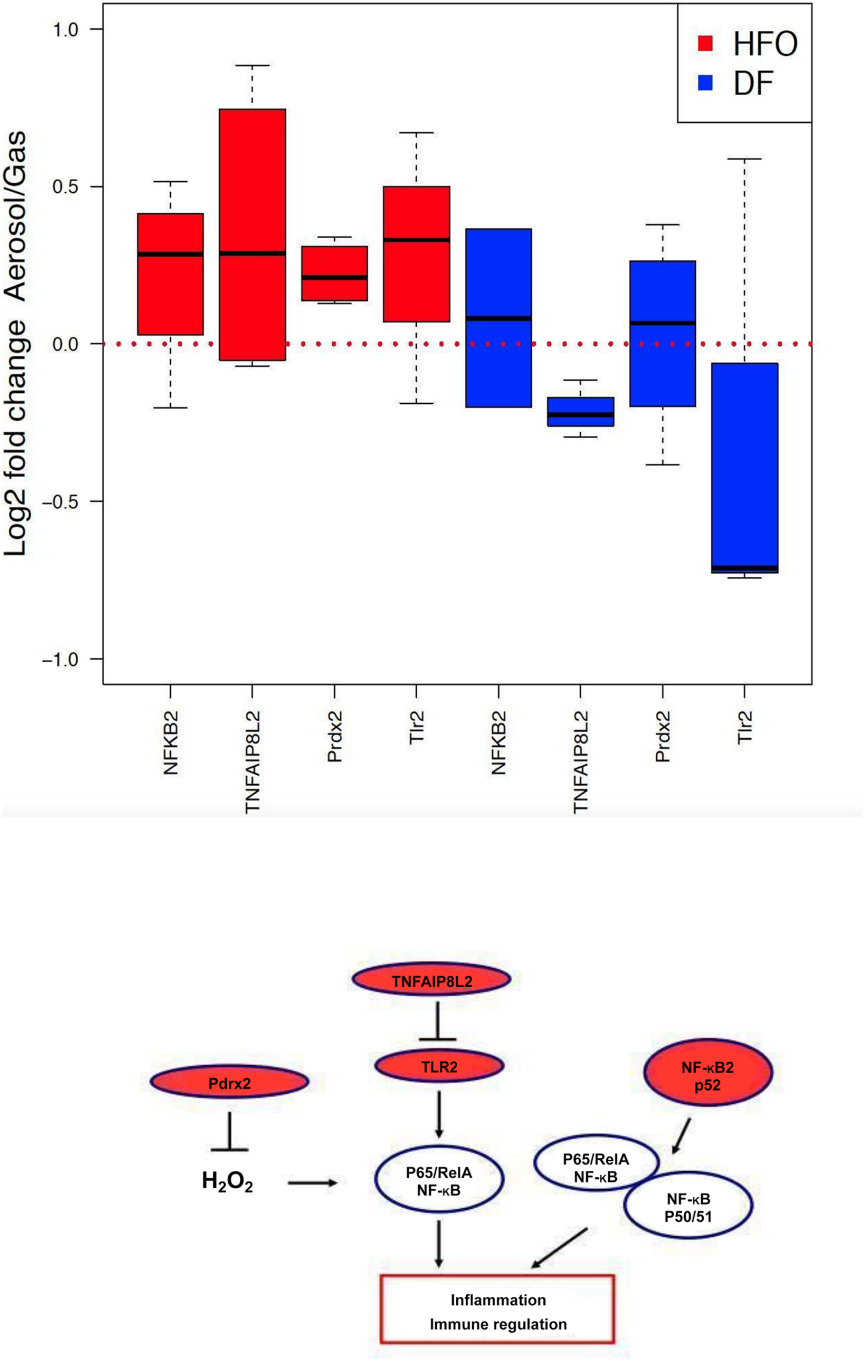
HFO particles induce activation of immune response in RAW 264.7 macrophages. (a) The Gene Ontology term GO:0006955, corresponding to activation of immune response, was found to be significantly up-regulated in HFO-treated samples (p = 0.059) and not regulated in the DF-treated samples. (b) Model of how the regulated proteins found in this study affect the NF-kB immune response pathway in the cell. Stimulation of the toll-like receptor (TLR2) leads to activation of NF-kB. Tumor necrosis factor alpha-induced protein 8-like protein 2 (TNFAIP8L2) acts as a negative regulator of TLR2, preventing hyperresponsiveness of the immune system, and inhibiting NF-kappa-B activation. Peroxiredoxin 2 (Pdrx2) reduces hydrogen peroxide, inhibiting NF-kappa-B activation.

Proteins relating to the GO term endocytosis (GO:0006897) were found to be upregulated in both HFO and DF treated samples, while the different fuel types induced activation of different groups of proteins involved in endocytosis (S8 Fig). Proteins involved in the immune response pathway (GO:0006955) were found to be upregulated only in HFO treated macrophages (p = 0.059), while DF stimulation shows no significant upregulation of the pathway (Fig 6). This data supports the metabolic analysis in so far that enrichment of the immune response pathway GO term indicates an activation of inflammatory pathways on a larger scale, mainly through the NF-kappa-B (NF-kB) signaling pathway (Fig 6).

Stimulation of the toll-like receptor (TLR2) leads to activation of NF-kB, which plays a key role in regulating the immune response. Tumor necrosis factor alpha-induced protein 8-like protein 2 (TNFAIP8L2) acts as a negative regulator of innate and adaptive immunity by maintaining immune homeostasis. TNFAIP8L2 prevents hyperresponsiveness of the immune system by negative regulation of TLR2, and inhibition of NF-kappa-B activation [48]. Peroxiredoxin 2 (Pdrx2) reduces hydrogen peroxide and is involved in redox regulation of the cell. Prdx2 inhibits NF-kB activation, which is induced by H_2_O_2_, and regulates immune response [49].

These findings in macrophages are in line with the previous study on lung epithelial cells [14]: the endocytosis pathway was found to be upregulated after aerosol exposure from both fuel types, while the immune response was only upregulated after HFO exposure.

## Discussion

Results from this study point to a differential effect between the gas and particle phases of combustion aerosols from ship engines on RAW 264.7 macrophages. For both of the fuel types studied, the emitted particle phase has a strong impact on cytotoxicity, while the gas phase of the aerosol alone has a stronger effect on the internal metabolism of the cells. The stronger gas phase effects seen with HFO aerosol suggest that the source of these effects could be from the compound class of smaller alkylated polycyclic aromatic hydrocarbons, such as alkylated phenanthrenes. These compounds are the only more abundant compound class detected in the HFO aerosol gas phase, if the (at the applied dilution) toxicologically noncritical sulfur dioxide is neglected. While this cannot be directly proven by this study, the results here motivate further experiments with semi-volatile compounds, such as smaller alkylated aromatics, to determine what, if any, gas phase effects are caused by their presence.

As different cellular effects can be seen due to gas exposure compared to combined gas and particle exposure, currently used submerged cell culture particle exposure experiments only partially highlight aerosol-induced changes in cellular metabolism; there should be more of a focus on research done with complete aerosol exposure in order to elucidate biological effects which are closer to those found *in vivo*.

The findings from this study validate and confirm the rather surprising results of the previously reported lung epithelial cell based multi-omics experiments on the effect of shipping engines emissions on lung epithelial cells [14], and extend the findings to a completely different cell type. This includes the observation that, according to the chemical analysis, the supposedly much less toxic particles from high quality, refined diesel fuel (DF) induce a broader biological reaction in lung cells than heavy fuel oil (HFO) particles at comparable exposure doses, although the toxicant concentration in particles from the latter is extremely high. As the overall particle mass deposited on the cells from DF exposure was lower than that from HFO, and the toxicity was around the same for both fuel types, it follows that DF exhaust is more toxic than was previously thought, and possibly on par with HFO. It was concluded that the high concentration of soot-like, elemental carbon in DF likely is responsible for this surprising effect [14]. As in the previous study with epithelial cells, a broader proteomic response after DF aerosol exposure was also seen in the macrophages, whereas HFO aerosol exposure was found to be an important inflammatory instigator, both on a metabolite and protein level. Endocytosis, which was identified as a regulated pathway in the epithelial cells, is also upregulated with exposure from both fuel types in macrophages, which is likely associated with particle uptake (S8 Fig).

Furthermore, it was observed in the previous study [14] that DF and HFO particles induce different biological pathways, assuming a different mode of action for the fairly different particle types (i.e. DF particles consist of carbon rich soot-like particle agglomerates while HFO particles are smaller, with mixed carbon/metal oxide cores and a surface covered with organic compounds and sulfates). The applied exposure concentrations (which are the same as in this study) were too low to induce direct cytotoxic effects in the epithelial cells. The macrophages, however, represent the first line of defense against particles and other pathogens in the lung. They can readily engulf the deposited particles through phagocytosis and thus are more vulnerable to PM exposure than lung epithelial cells. The cytotoxicity results summarized in Fig 2 suggest that the DF particles exhibit a stronger cytotoxicity than the particles from the HFO aerosol. This lends further evidence to the unexpected result from the previous study, that cleaner fuels (i.e. fuels that reduce the emission of toxic chemicals) do not automatically implicate a lower acute cytotoxicity of the formed particles.

Previous studies using submerged culture exposure of collected particles show induction of cytotoxicity and inflammatory signals in epithelial cells as well as macrophages [4, 6]. Our results, determined using the more realistic air-liquid interface cell exposure, support these findings by adding metabolic inflammatory signals found under aerosol exposure. However, it becomes clear from our data that the strength of inflammatory signals depend on the specific properties and source of the aerosol, and that this effect is not only dependent on particle exposure alone. HFO aerosols stimulate a pro-inflammatory response in the macrophages, leading to an increase in metabolites and protein regulatory pathways known to be associated with antimicrobial activity and a pro-inflammatory phenotype. This is especially highlighted by the induction of a metabolite indicating inflammation, itaconic acid, by HFO exposure. Proteomics data supports this finding by showing an upregulation of inflammatory-associated proteins in the NF-kB signaling pathway. This phenotypic effect is solely induced by the gas phase of the combustion aerosol. The increase of succinic acid levels in macrophages suggest a role for HIF-1a in this process, and these signaling pathways would be promising for continued research into aerosol-based inflammation in macrophages.

While air-liquid interface exposures already represent a step towards a more realistic model of airway exposure, an important further improvement would be taking into account the pulmonary surfactant system, integrating it into an improved air-liquid interface exposure system. It has been shown that proteins from this system have an impact on how pathogen-associated molecular pattern recognizing proteins and cytokines are expressed in stimulated macrophgaes [50], and addition of this system into the experimental setup used in this study would thus increase it’s applicability even further.

As metabolism is becoming more and more implicated in the inflammatory response of many cells (including macrophages), as well as the progression of many diseases (including cardiovascular diseases and cancer), studies integrating metabolomics with other techniques (such as proteomics), probing the effect of anthropogenic aerosols on metabolism, are vital to increase our understanding of cellular mechanisms underlying the negative health effects. Furthermore, a focus should be put on studies that characterize the complete aerosols found in the environment, with in-depth research as well on the possible effects of gaseous components on human health. In this way, the complex composition of aerosols can be broken down, and simpler models of aerosol can be studied to understand the most important components of aerosol relative to human health effects. Finally, it can be seen that fuel changes alone are not sufficient for the mitigation of health effects from aerosol combustion, and means of reducing particles should be integrated into the reduction of shipping-related health effects.

Finally, this work support the conclusion of the previous work [14], that legislation enforcing fuel changes (i.e. establishment of sulphur emissions control areas, SECA [51] alone are not sufficient for a safe mitigation of health effects from shipping aerosol emissions. Moreover, measures reducing particles and toxic gases from shipping emissions (exhaust gas scrubbers, particle filters, precipitators, or others) need to be further developed and legally prescribed for the reduction of shipping-related health effects.

## Supporting Information

S1 File Previous Supporting Work. The manuscript Oeder *et al* 2015, which is referenced by this work.
(PDF)

S1 Table Metabolite List. List of final 34 metabolites identified in experimental intracellular samples. All compounds were identified with an in-house library.
(EPS)

S2 Table Metabolomcs Dataset. Non-targeted metabolomics dataset containing the 230 compounds found in each of the four different experimental conditions.
(CSV)

S3 Table LDH Dataset. Raw data from the lactate dehydrogenase (LDH) assay.
(CSV)

S1 Fig Chemical Characterization of Particulate Matter In Aerosol. Concentrations (right) and concentration ratios (left) of particulate matter-bound species in HFO and DF aerosols. Modified from Oeder et al, 2015. Exponents refer to methods used to obtain data: (5) ICP-AES, (6) Thermal desorption/direct derivatization gas chromatography/Mass spectrometry, (7) AMS, (8) Filter weighing, (9) EC/OC-analysis (thermal-optical method), (10) Aethalometer.
(EPS)

S2 Fig Itaconic Acid Identification. Mass spectrum of experimentally discovered itaconic acid (top) compared with the in-house library spectrum (bottom). Retention index of experimental compound was 1417.21, while retention index of library compound is 1409.20. Using the ICBM algorithm for matching, experimental itaconic acid was identified with a score of 0.97.
(EPS)

S3 Fig Proteome Christmas Tree Plot. Comparison of regulation magnitude and abundance of regulated proteins. Mean of log2 fold change Aerosol vs. Gas is plotted vs. mean of log10 fold sum of intensities of complete dataset of proteins in response to DF (blue) and HFO (red) particles. RAW 264.7 cells show broadening of the protein regulation in response to DF particles (blue) in comparison to HFO particles (red), corresponding to higher amount of up and down regulated proteins in DF particles treated samples.
(EPS)

S4 Fig Proteome Volcano Plot. Comparison of regulation magnitude and regulation significance. Mean of log2 fold change Aerosol vs. Gas is plotted vs. -log10 p-value of complete dataset of proteome in response to DF (blue) and HFO (red) particles. RAW 264.7 cells show broadening of the significant protein regulation in response to DF particles (blue) in comparison to HFO particles (red), corresponding to higher amount of significantly up and down regulated proteins in DF particles treated samples.
(EPS)

S5 Fig Proteome Correlation Plot. Correlation of log2 fold changes Aerosol vs. Gas between HFO and DF. Log2 fold changes Aerosol vs. Gas of protein regulation in response to HFO and DF show no correlation (Cor2 = 00357), which indicates that HFO and DF particles cause different regulation of different proteins in RAW 264.7 cells.
(EPS)

S6 Fig Proteome QQ Plot—DF. The Q–Q plot for proteomic response of RAW 264.7 cells to DF Aerosol vs. DF Gas. The line is a parametric curve with the parameter, which is the interval for the quantile. The points with linearly related distributions lie on the line, which suggests that they are normally distributed. On the left and right sides of the plot the points are not linearly related and show no normal distribution, which corresponds to populations of up and down regulated proteins in response to DF particles.
(EPS)

S7 Fig Proteome QQ Plot—HFO. The Q–Q plot for proteomic response of RAW 264.7 cells to HFO Aerosol vs. HFO Gas. The line is a parametric curve with the parameter, which is the interval for the quantile. The points with linearly related distributions lie on the line, which suggests that they are normally distributed. On the left and right sides of the plot the points are not linearly related and show no normal distribution, which corresponds to populations of up and down regulated proteins in response to HFO particles.
(EPS)

S8 Fig Regulation of Endocytosis Related Proteins. DF and HFO particles induce activation of different proteins involved in endocytosis in RAW 264.7 macrophages. The Gene Ontology term Endocytosis was found up regulated in HFO and DF Aerosol vs. Gas treated samples. This data suggests that particles play a role in the regulation of endocytosis in macrophages. Necap1, Necap2 and Lrp1 (red) are up regulated in HFO treated samples, while Lrp1, Vamp7, Tfrc and Ube3a (blue) are up regulated in DF treated samples.
(EPS)
